# Supplementary material for: Genome-wide identification, characterization and gene expression of BES1 transcription factor family in grapevine (Vitis vinifera L.)
Source: Sci Rep. 2023 Jan 5;13:240. doi: 10.1038/s41598-022-24407-y (PMC9816167; doi:10.1038/s41598-022-24407-y)
Supplement: Supplementary file 3 — Supplementary Information. [file 41598_2022_24407_MOESM3_ESM.zip › Vvi_Atr/Vitis_vinifera.PN40024.v4.dna_sm.toplevel.fa.vs.Amborella_trichopoda.AMTR1.0.dna_sm.toplevel.fa.html/Atr-AmTr_v1.0_scaffold00177.html]

|  |  |  |  |  |  |  |  |  |  |  |  |  |  |
| --- | --- | --- | --- | --- | --- | --- | --- | --- | --- | --- | --- | --- | --- |
| Duplication depth | Reference chromosome | Collinear blocks | | | | | | | | | | | |
| 0 | Atr-ERN10281 |  |  |  |  |  |  |  |  |  |  |  |  |
| 0 | Atr-ERN10282 |  |  |  |  |  |  |  |  |  |  |  |  |
| 0 | Atr-ERN10283 |  |  |  |  |  |  |  |  |  |  |  |  |
| 2 | Atr-ERN10284 |  | Vvi-Vitvi01g00080\_t001 |  | Vvi-Vitvi14g02014\_t001 |  |  |  |  |  |  |  |  |
| 2 | Atr-ERN10285 |  | \| \| |  | \| \| |  |  |  |  |  |  |  |  |
| 2 | Atr-ERN10286 |  | \| \| |  | \| \| |  |  |  |  |  |  |  |  |
| 2 | Atr-ERN10287 |  | \| \| |  | \| \| |  |  |  |  |  |  |  |  |
| 2 | Atr-ERN10288 |  | \| \| |  | \| \| |  |  |  |  |  |  |  |  |
| 2 | Atr-ERN10289 |  | \| \| |  | \| \| |  |  |  |  |  |  |  |  |
| 2 | Atr-ERN10290 |  | Vvi-Vitvi01g00077\_t001 |  | \| \| |  |  |  |  |  |  |  |  |
| 2 | Atr-ERN10291 |  | \| \| |  | Vvi-Vitvi14g02015\_t001 |  |  |  |  |  |  |  |  |
| 2 | Atr-ERN10292 |  | \| \| |  | \| \| |  |  |  |  |  |  |  |  |
| 2 | Atr-ERN10293 |  | Vvi-Vitvi01g00076\_t002 |  | \| \| |  |  |  |  |  |  |  |  |
| 2 | Atr-ERN10294 |  | Vvi-Vitvi01g00075\_t001 |  | \| \| |  |  |  |  |  |  |  |  |
| 2 | Atr-ERN10295 |  | Vvi-Vitvi01g00074\_t001 |  | \| \| |  |  |  |  |  |  |  |  |
| 2 | Atr-ERN10296 |  | \| \| |  | Vvi-Vitvi14g03097\_t001 |  |  |  |  |  |  |  |  |
| 2 | Atr-ERN10297 |  | \| \| |  | \| \| |  |  |  |  |  |  |  |  |
| 2 | Atr-ERN10298 |  | \| \| |  | \| \| |  |  |  |  |  |  |  |  |
| 2 | Atr-ERN10299 |  | \| \| |  | \| \| |  |  |  |  |  |  |  |  |
| 2 | Atr-ERN10300 |  | Vvi-Vitvi01g01846\_t001 |  | \| \| |  |  |  |  |  |  |  |  |
| 1 | Atr-ERN10301 |  |  |  | \| \| |  |  |  |  |  |  |  |  |
| 1 | Atr-ERN10302 |  |  |  | \| \| |  |  |  |  |  |  |  |  |
| 1 | Atr-ERN10303 |  |  |  | \| \| |  |  |  |  |  |  |  |  |
| 1 | Atr-ERN10304 |  |  |  | \| \| |  |  |  |  |  |  |  |  |
| 1 | Atr-ERN10305 |  |  |  | Vvi-Vitvi14g02016\_t001 |  |  |  |  |  |  |  |  |
| 1 | Atr-ERN10306 |  |  |  | \| \| |  |  |  |  |  |  |  |  |
| 1 | Atr-ERN10307 |  |  |  | \| \| |  |  |  |  |  |  |  |  |
| 1 | Atr-ERN10308 |  |  |  | Vvi-Vitvi14g04693\_t001 |  |  |  |  |  |  |  |  |
| 1 | Atr-ERN10309 |  |  |  | \| \| |  |  |  |  |  |  |  |  |
| 1 | Atr-ERN10310 |  |  |  | \| \| |  |  |  |  |  |  |  |  |
| 1 | Atr-ERN10311 |  |  |  | \| \| |  |  |  |  |  |  |  |  |
| 1 | Atr-ERN10312 |  |  |  | Vvi-Vitvi14g02018\_t001 |  |  |  |  |  |  |  |  |
| 1 | Atr-ERN10313 |  |  |  | \| \| |  |  |  |  |  |  |  |  |
| 1 | Atr-ERN10314 |  |  |  | \| \| |  |  |  |  |  |  |  |  |
| 1 | Atr-ERN10315 |  |  |  | \| \| |  |  |  |  |  |  |  |  |
| 1 | Atr-ERN10316 |  |  |  | Vvi-Vitvi14g02020\_t001 |  |  |  |  |  |  |  |  |
| 1 | Atr-ERN10317 |  |  |  | Vvi-Vitvi14g02021\_t001 |  |  |  |  |  |  |  |  |
| 1 | Atr-ERN10318 |  |  |  | Vvi-Vitvi14g02022\_t001 |  |  |  |  |  |  |  |  |
